# Colopancreatic Fistula: An Uncommon Complication of Recurrent Acute Pancreatitis

**DOI:** 10.1155/2018/4521632

**Published:** 2018-03-27

**Authors:** Mouhanna Abu Ghanimeh, Omar Abughanimeh, Khalil Abuamr, Osama Yousef, Esmat Sadeddin

**Affiliations:** ^1^Henry Ford Health System, 2799 W Grand Blvd, Gastroenterology K-7 Room E-744, Detroit, MI 48202, USA; ^2^Internal Medicine, Graduate Medical Education, School of Medicine, University of Missouri-Kansas City, 2411 Holmes Street, M2-302, Kansas City, MO 64108, USA; ^3^Gastroenterology, School of Medicine, University of Missouri-Kansas City, 2411 Holmes Street, Kansas City, MO 64108, USA

## Abstract

Colonic complications, including colopancreatic fistulas (CPFs), are uncommon after acute and chronic pancreatitis. However, they have been reported and are serious. CPFs are less likely to close spontaneously and are associated with a higher risk of complications. Therefore, more definitive treatment is required that includes surgical and endoscopic options. We present a case of a 62-year-old male patient with a history of heavy alcohol intake and recurrent acute pancreatitis who presented with a 6-month history of watery diarrhea and abdominal pain. His abdominal imaging showed a possible connection between the colon and the pancreas. A further multidisciplinary workup by the gastroenterology and surgery teams, including endoscopic ultrasound, endoscopic retrograde cholangiopancreatography, and colonoscopy, resulted in a diagnosis of CPF. A distal pancreatectomy and left hemicolectomy were performed, and the diagnosis of CPF was confirmed intraoperatively. The patient showed improvement afterward.

## 1. Introduction

Acute and chronic pancreatitis are not uncommon [1–3]. Both conditions can be associated with a variety of systemic as well as local complications [3–7]. Colonic complications are uncommon with acute and chronic pancreatitis. However, when they occur, they are usually serious and require further intervention [5–7]. These complications range from a localized ileus to severe colonic ischemia and necrosis with resulting perforation, hemorrhage, or fistula formation [5–9]. Colopancreatic fistulas (CPFs) occur in 3* *%* *–* *10* *% of patients with severe acute pancreatitis and can occur with chronic pancreatitis as well [5, 8–10]. When CPFs are suspected, computed tomography (CT) scan of the abdomen with or without contrast is a useful initial diagnostic tool [11]. Colonoscopy might also detect many cases [9]. As CPFs are less likely to close spontaneously and are usually associated with a complicated course, a more definitive intervention is typically warranted [5, 8]. Treatment modalities include surgical as well as endoscopic options [12–15].

## 2. Case Report

A 62-year-old male patient with a history of heavy alcohol intake and recurrent episodes of acute pancreatitis presented with a 6-month history of watery diarrhea, abdominal pain, weight loss and severe electrolyte abnormalities.

The patient had had an extensive workup in the last 3 months prior to presentation. His tissue transglutaminase IgA was 1 unit/ml (normal: < 4 units/ml), stool studies showed normal split fat and were negative for* Clostridium difficile, *ova, and parasites, esophagogastroduodenoscopy (EGD) showed mild gastritis with negative biopsies for* Helicobacter pylori,* and colonoscopy showed uncomplicated internal hemorrhoids. At that time, these tests did not reveal a significant pathology to explain his symptoms.

Upon evaluation, he appeared to be dehydrated and distressed. An abdominal examination showed mild tenderness to palpation with no rebound tenderness, guarding, or rigidity.

The initial laboratory workup showed the following levels: Na 131 mmol/L (normal: 136–144 mmol/L), Cl 94 mmol/L (normal: 95–105 mmol/L), K 3.0 mmol/L (normal: 3.6–5.1 mmol/L), creatinine 1.5 mg/dl (normal: 0.9–1.2 mg/dl), lipase 81 units/L (normal: 13–60 units/L), hemoglobin 10.9 g/dl (normal: 12–16 g/dl), and WBC 9,600/cmm (normal: 4,000-11,000/cmm). The patient was admitted for fluid and electrolyte replacement, symptomatic control, and additional evaluation of his underlying symptoms.

**Figure 1:**
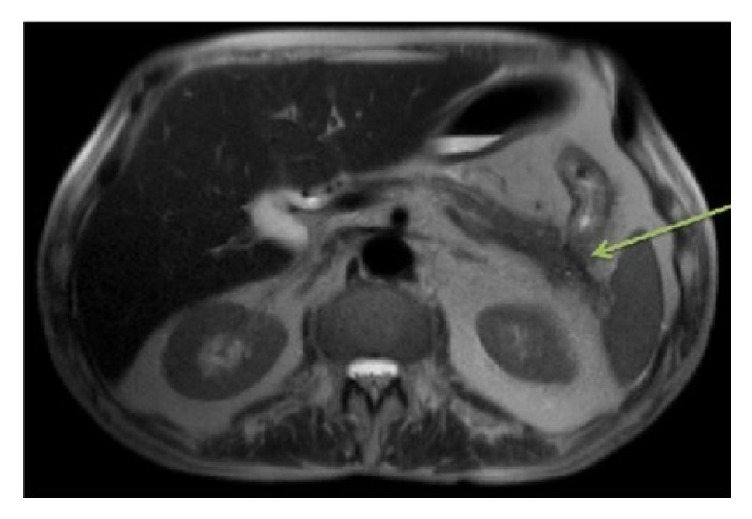
MRCP showing cystic foci in the pancreatic tail and colonic wall thickening. No strictures were noted. Green arrow denotes possible connection between the colon and pancreas.

The CT scan of the abdomen without contrast showed a prominent common bile duct (CBD), dilated gallbladder (GB), and colonic wall thickening that was not thought to be significant initially. Magnetic resonance cholangiopancreatography (MRCP) was performed later (given the abnormalities in GB and CBD) and excluded strictures in the CBD. However, it did show cystic foci in the pancreatic tail and persistent colonic wall thickening [Figure 1]. This finding permitted greater discussion regarding endoscopic ultrasound (EUS) and possible endoscopic retrograde cholangiopancreatography (ERCP).

**Figure 2:**
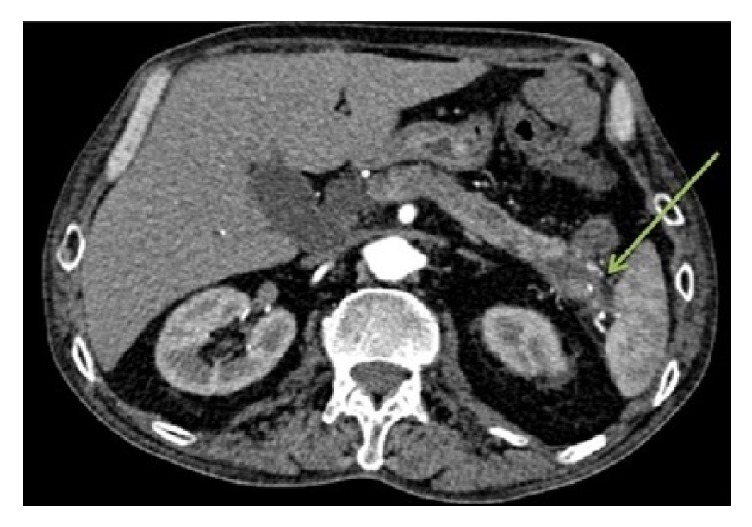
CTA showing a pancreatic tail lesion, with a focus of fluid and interval diffuse progression of colonic wall thickening. The pancreatic tail was inseparable from the colon, with a possible connection between the colon and the pancreas. Green arrow denotes possible connection between the colon and pancreas.

Prior to the EUS and ERCP, a CT scan angiography of the abdomen (CTA) was performed to rule out mesenteric ischemia. The pancreatic tail lesion was remarkable, with an internal focus of fluid and interval diffuse progression of circumferential colonic wall thickening. The pancreatic tail was inseparable from the colon, with a possible connection between the colon and the pancreas [Figure 2]. The tumor marker CA 19-9 was 36.6 units/ml (normal: <37 units/ml).

**Figure 3:**
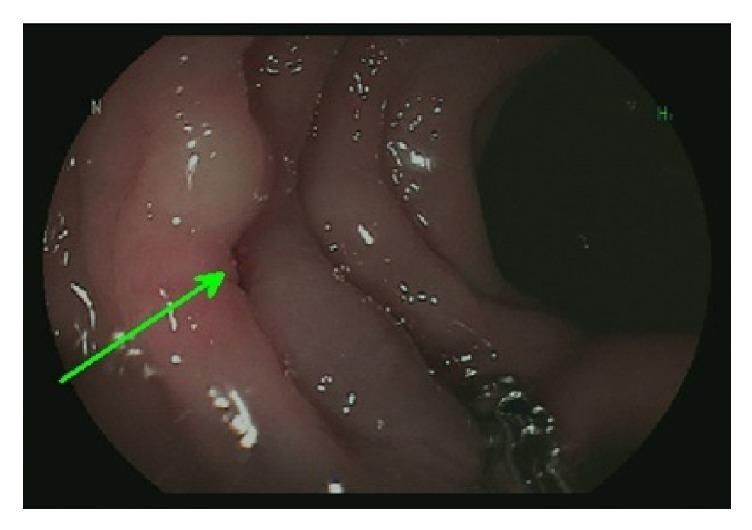
Colonoscopy showing the localized edematous area with bluish discoloration 45 cm from the anus that correlated with the area revealed in the CT scan. Green arrow denotes the edematous area on colonoscopy about 45 cm from anus which likely represents fistula's opening.

An EUS showed an anechoic lesion that was 10 × 11 mm, a possible cystic structure in the pancreatic tail with mild hypoechogenicity in the surrounding parenchyma. Fine-needle aspiration (FNA) was done and revealed ductal and inflammatory cells with Gram-negative rods. This finding increased the suspicion of a colonic connection. A repeated colonoscopy [Figure 3] was significant for a localized edematous area with bluish discoloration 45 cm from the anus that correlated with the area revealed in the CT scan. The surgery team was consulted, and an ERCP was scheduled to define the anatomy of the area, especially preoperatively.

The ERCP did not show an extravasation of contrast. The surgery team decided to proceed with surgery, as the patient continued to have symptoms. A pancreatectomy, splenectomy, and partial left colectomy were performed. It was possible to visualize the posterior colonic surface near the splenic flexure intraoperatively, which showed a connection between the colon and the pancreas. The patient showed improvement postoperatively, with complete resolution of his symptoms. He continued to attend surgery and gastroenterology clinics.

## 3. Discussion

Acute pancreatitis is an acute inflammatory condition of the pancreas. It is characterized by abdominal pain and elevated serum levels of pancreatic enzymes, primarily amylase and lipase [1]. Chronic pancreatitis is an enduring and progressive fibroinflammatory process that eventually leads to permanent pancreatic structural damage, resulting in exocrine and endocrine dysfunction [2].

Acute pancreatitis is not uncommon, with a reported annual incidence ranging from 4.9 to 35 per 100,000 individuals [3]. The overall mortality rate of acute pancreatitis is approximately 5%. Acute necrotizing pancreatitis usually is associated with a worse prognosis than acute interstitial pancreatitis. The mortality rate is 17% versus 3%, respectively [4].

Colonic complications are uncommon with acute and chronic pancreatitis [5–7]. However, they have been reported and are potentially serious. Different studies have shown an incidence of 3.3%–6.1% of acute pancreatitis [5, 8]. The incidence increased to 15% among patients with acute severe pancreatitis [5]. These complications are of variable severity. They include but are not limited to a localized ileus, obstruction due to severe edema or inflammation, colonic ischemia with or without necrosis, hemorrhage, and fistula formation [5, 7].

CPFs occur in 3* *%* *–* *10* *% of patients with severe acute pancreatitis [5, 8–10]. They can occur with chronic pancreatitis as well [8]. The time course between acute pancreatitis and CPF formation is variable, ranging from 10 to 180 days, according to different studies [5, 16]. Due to the anatomical proximity, the left side of the colon is more commonly involved in CPFs [17].

The clinical presentation of CPFs is either colonic symptoms such as bleeding or diarrhea or common symptoms of acute pancreatitis such as pain, nausea, and vomiting [5, 9]. Gastrointestinal bleeding is one of the most common presentations of CPFs, with 60% of patients presenting with it [18].

CT scan of the abdomen with or without contrast is useful in the diagnosis of CPFs and all pancreatic-enteric fistulas in general [11]. Additionally, colonoscopy usually detects many cases, including the patient in this report. However, the patient could have numerous fistulas at the time of surgery [9].

In contrast to upper pancreatic-enteric fistulas, which may close spontaneously, CPFs generally are less likely to close spontaneously [5, 8]. They are associated with a higher risk of complications, including spontaneous or persistent infection, severe hemorrhage, perforation, or overwhelming infections [5, 8]. Therefore, more definitive treatment is recommended, typically with surgery [5, 6, 8, 10].

Surgery usually involves resection of the involved part of the colon with or without a distal pancreatectomy. A diverting ileostomy, to preserve the colon, was reported in less severe cases [5, 6, 10]. Drainage of any abscess in the peripancreatic area or around the fistula is also necessary [10, 16].

Less invasive interventions such as endoscopic treatment have been described in some cases [12–15, 19–23]. However, there are no clear guidelines for the use of these techniques as standard treatment until now [13]. Fibrin glue alone can be used, especially in small fistulas [24]. The use of hemoclips, either via the conventional hemoclips or via an over-the-scope clip system, were described in many case reports with favorable outcomes [13, 19, 20]. Over the scope clip system is a technique that involves mechanical compression of the gastrointestinal tissue which facilitates tissue entrapment [21]. It was introduced by Kirschniak et al. [22] and has been used in management of gastrointestinal bleeding, compression of large vessels, and closure of defects along the gastrointestinal tract [21]. In general, these endoscopic techniques are more suitable to close small defects <10 mm in diameter [19]. Will et al. [23] described a unique endoscopic technique to close the colonic end of the fistula with ligation bands, endoloops, fibrin glue, and hemoclips. This technique involves an endoscopic rendezvous that approached both ends of the fistula simultaneously.

In our case, the radiological evidence of colonic wall thickening and the proximity between the pancreases and the colon raised the suspicion of CPFs. This was supported by the growth of enteric Gram-negative rods in the FNA and the colonoscopic findings. The challenging point in the diagnosis was the failure of the ERCP to show the extravasation of the contrast. Nevertheless, we think that this can be explained by the complexity in the anatomy of the fistula that prevented the contrast from passing. In this case, the multidisciplinary decision was made to proceed with surgery, given the clinical symptoms and all the evidence described above. The direct visualization of the communication between the pancreas and the colon intraoperatively added additional evidence of the presence of CPF. The standard surgical intervention was performed based on the availability of resources and expertise. Hopefully, clear guidelines can be developed so that patients do not have to undergo these invasive procedures.

## 4. Conclusion

Colonic complications, including CPFs, are uncommon with acute and chronic pancreatitis. However, they are reported and potentially serious. A definitive treatment, typically with surgery, is usually recommended. Less invasive interventions such as endoscopic treatment have been described as well.
